# Differential Dependence on Host Cell Glycosaminoglycans for Infection of Epithelial Cells by High-Risk HPV Types

**DOI:** 10.1371/journal.pone.0068379

**Published:** 2013-07-04

**Authors:** Linda Cruz, Craig Meyers

**Affiliations:** Department of Microbiology and Immunology, The Pennsylvania State University College of Medicine, Hershey, Pennsylvania, United States of America; Johns Hopkins School of Medicine, United States of America

## Abstract

Human papillomavirus (HPV) infection is the leading cause of cervical cancer world-wide. Here, we show that native HPV particles produced in a differentiated epithelium have developed different strategies to infect the host. Using biochemical inhibition assays and glycosaminoglycan (GAG)-negative cells, we show that of the four most common cancer-causing HPV types, HPV18, HPV31, and HPV45 are largely dependent on GAGs to initiate infection. In contrast, HPV16 can bind and enter through a GAG-independent mechanism. Infections of primary human keratinocytes, natural host cells for HPV infections, support our conclusions. Further, this renders the different virus types differentially susceptible to carrageenan, a microbicide targeting virus entry. Our data demonstrates that ordered maturation of papillomavirus particles in a differentiating epithelium may alter the virus entry mechanism. This study should facilitate a better understanding of the attachment and infection by the main oncogenic HPV types, and development of inhibitors of HPV infection.

## Introduction

Human papillomavirus (HPV) is the causative agent of cervical cancer and other anogenital cancers and oropharyngeal cancers [1], [2]. The major capsid protein, L1, mediates primary attachment of viral particles to cells [3] and the extracellular matrix [4]. The minor capsid protein, L2, is essential for infection, having multiple roles in genome encapsidation, capsid stabilization, receptor-binding, endosomal escape, and escorting the viral genome to the nucleus [5]–[11]. Current papillomavirus vaccines target the major capsid protein L1 of the most common cancer-causing types, HPV16 and HPV18, which together account for 70% of cervical cancer cases, protecting against virus infection and development of neoplasias [12]. However, since current vaccines are type-specific, they do not offer protection against all cancer-causing HPV types. In addition, they are cost-prohibitive to most women around the world [13], [14]. Thus, there is a need for the development of less expensive alternatives, such as universal microbicides in addition to the current vaccines.

HPV infects basal keratinocytes and the production of new particles is closely tied to the cellular differentiation pattern of epithelial cells. The complete HPV life cycle can be recapitulated *in vitro* in organotypic raft culture [15]–[18]. Thus far, infection and entry studies have mostly been done using pseudovirions (PsV), which are efficiently produced by over-expression and self-assembly of the capsid proteins in monolayers [19]. Using this system, most papillomaviruses have been observed to infect cells by first attaching to a form of glycosaminoglycan (GAG), heparin sulfate (HS), via L1 to the cell surface or extracellular matrix (ECM) [3], [20], [21]. Initial binding to laminin-332 (laminin 5) on the ECM has also been demonstrated [22], [23]. HS attachment induces a conformational change allowing for the L2 N-terminus to be cleaved by a proprotein convertase (PC), furin and/or PC5/6 [24], [25]. Following HS attachment and cleavage of L2 by furin and/or PC5/6, the virus is thought to be transferred to a secondary entry receptor [26], [27]. Alpha6-integrin, growth factor receptors, and annexin A2 have been suggested as potential candidate receptors, however their role in infection is still unclear [11], [28], [29]. The conformational changes required for infectious entry of virus particles have been shown to be mediated by cyclophilin B for some, but not all, HPV PsV types tested [30]. Primary attachment to HS has been suggested to be a universal entry step for all papillomaviruses. However, noticeably, tissue-derived HPV31 native virus (NV) infection of human keratinocytes was shown not to require HS [31]. Furthermore, members of the closely related polyomavirus family have been shown to utilize different receptors [32]–[34]. Thus, a general hypothesis for HPV attachment and entry may not encompass all HPV types. In addition, while PsV has proven to be very important in the understanding of the process of HPV infection, it is not well understood what structural differences compared to authentic virions exist and how these structural alterations might affect the biology of the virus. In a study of the cross-neutralizing ability of neutralizing antibodies against L2 N-terminal epitopes, it was shown that there were differences in the neutralizing pattern of PsV as compared to NV particles, suggesting there may be overall structural differences between PsV and NV particles [35]. Consequently, structural differences between particles may impose functional differences on virus binding and infection.

To further the understanding of HPV entry, we set out to investigate whether diverse HPV types produced under physiologically relevant conditions of differentiating host tissue are dependent on GAG-mediated binding for infection. Here, we examined the requirement of the high-risk HPV types HPV16, HPV18, HPV31, and HPV45 for GAG binding during infection. Our results suggest that different HPV types show specific preferences for the type of GAG and the sulfation status of the GAGs for attachment and infection as seen with HPV18, HPV31, and HPV45. In contrast, HPV16 may infect their host cells independent of GAGs.

## Results

### Infection and Neutralization for Analyses of High-risk HPVs

**Figure 1:**
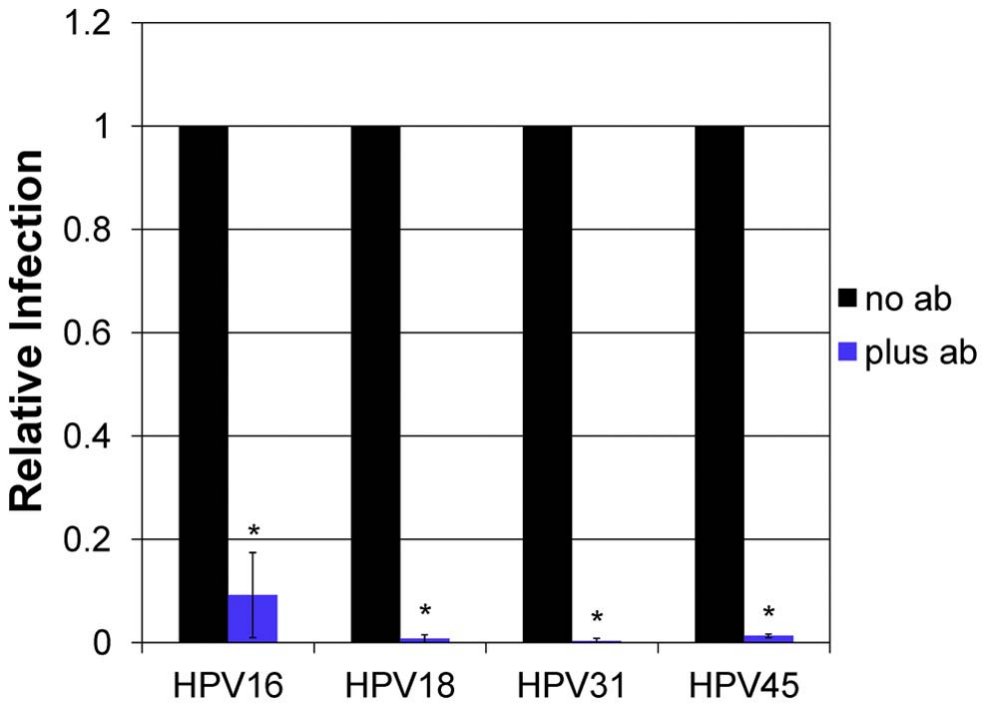
Particle-mediated infection. HPV16, HPV18, HPV31, and HPV45 were incubated with L1 type-specific antibodies H16.V5, H18.J4, H31.A6, and H45.N5, respectively, for 1 hour at 37°C prior to infection of HaCaT cells. Infections were analyzed by RT-qPCR measuring the relative amount of E1^E4 transcript two days post-infection.

The production and neutralization of the most common high-risk papillomaviruses HPV16, HPV18, and HPV45 in foreskin-derived organotypic raft culture has been previously shown [15]–[17]. Native HPV31 was produced from a cervical intraepithelial neoplasia type 1 biopsy-derived cell line CIN-612 9E [18]. To verify the specific particle-mediated infectious entry as well as the specificity of the RT-qPCR assay for the analysis of HPV infection, we neutralized each virus with a type-specific monoclonal antibody targeting a major epitope in the L1 major capsid protein (figure 1).

### Inhibitory Effects of the HS Mimetic Heparin

**Figure 2:**
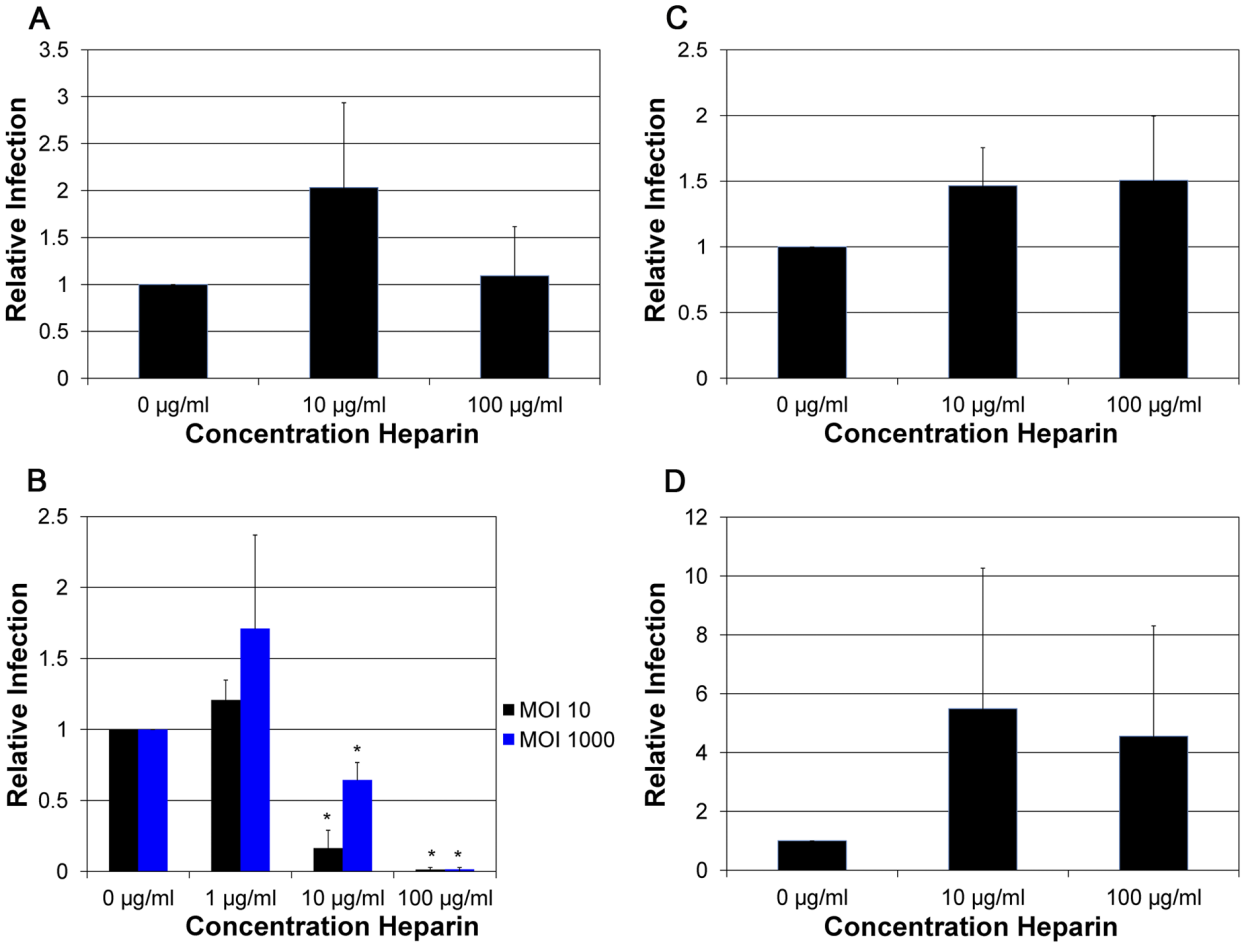
Inhibitory effects of heparin against various HPV types. A) HPV16, B) HPV18, C) HPV31, and D) HPV45 were incubated with heparin at increasing concentrations (0 µg/ml, 1 µg/ml, 10 µg/ml, and 100 µg/ml) for 30 min’s at 37°C prior to infection and during infection of HaCaT cells. All infections were done at an MOI of 10. For HPV18 additional infections at a MOI of 100 and 1000 were performed. Infections were analyzed by RT-qPCR measuring the relative amount of E1^E4 transcript two days post-infection. The data is plotted as relative infection at the different concentrations with infection at 0 µg/ml of heparin set equal to one.

Heparin, a highly sulfated form of HS produced from mast cells, is thought to bind to HPV particles and prevent binding to the cells [3]. HPV16 infection of HaCaT cells was not blocked by heparin (figure 2A). In contrast, infection by HPV18 was efficiently blocked by heparin in a dose-dependent manner (figure 2B). HPV31, which is evolutionary related to and found in the same species (α9) as HPV16, showed the same resistance to heparin as HPV16 (figure 2C). This confirms data from a previous study on infection by HPV31, where the presence of heparin had no effect on infection of HaCaT cells [31]. Infection by HPV45, which is related to and found in the same species (α7) as HPV18, was also resistant to inhibition by heparin (figure 2D). Thus, functional studies rather than sequence conservation and relative relatedness between different HPV types are necessary for the prediction of responsiveness to a given blocking agent. At a multiplicity of infection (MOI) of 10 particles, HPV18 infection was unaffected by the presence of 1 µg/ml heparin, with 90% inhibition observed at 10 µg/ml heparin (figure 2B). When infections were done with an MOI of 1,000 particles the inhibitory ability of heparin decreased, with 50% inhibition observed at 10 µg/ml heparin. This is in contrast to a PsV inhibition assay at a similar inoculum where a 10-fold lower concentration is needed for 50% inhibition of infection [13], suggesting that HPV18 NV particles are less sensitive to inhibition by heparin than PsV. The observed capsid dose-dependence of inhibition further supports the direct binding of heparin to the HPV18 NV particles.

### Heparin and Virus Attachment

**Figure 3:**
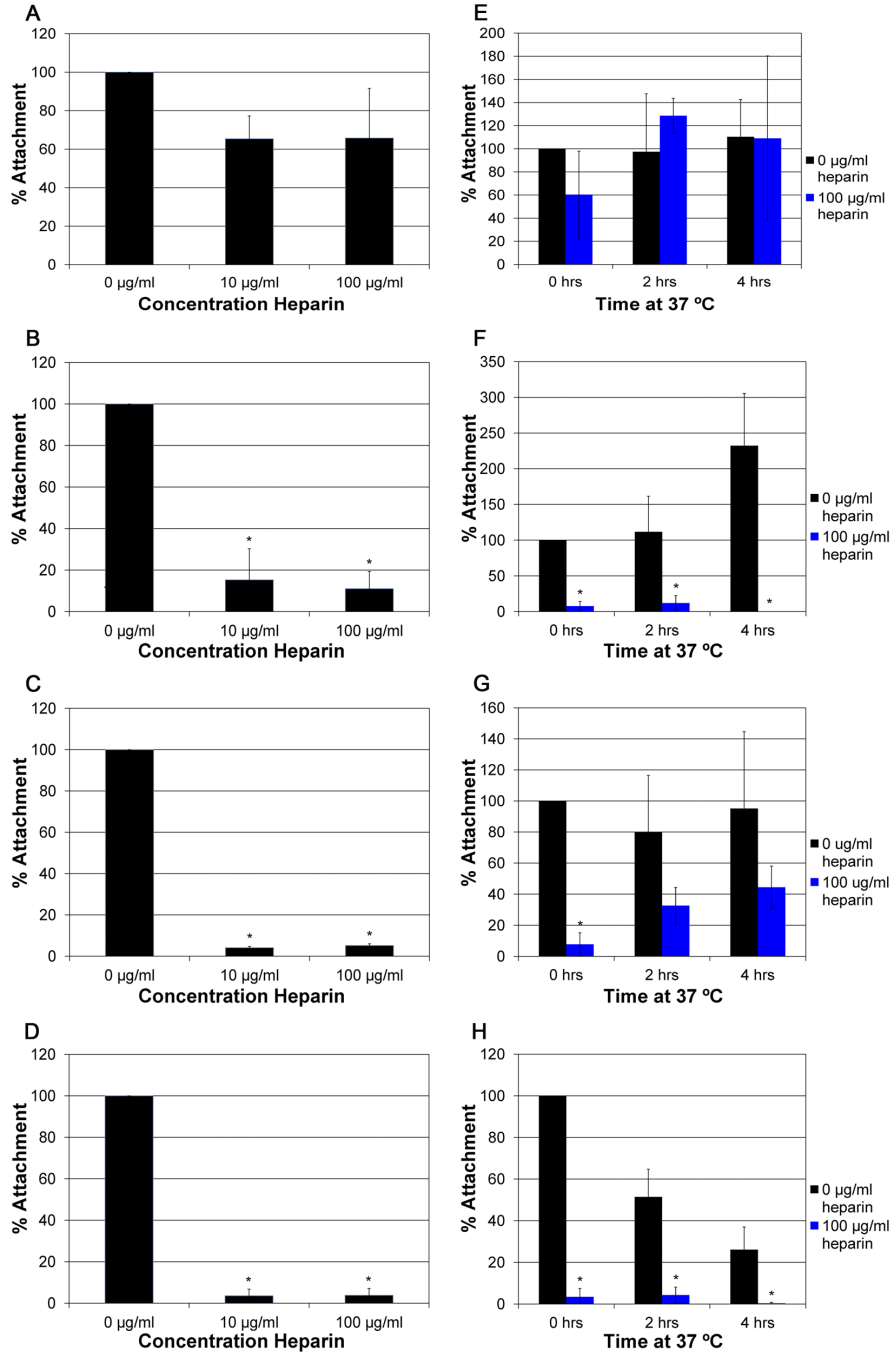
Effect on absorption in the presence of heparin. A) HPV16, B) HPV18, C)HPV31, and D) HPV45 were mixed with heparin at increasing concentrations (0 µg/ml, 10 µg/ml, and 100 µg/ml) and added to HaCaT cells at an MOI of 25. The virus was allowed to attach to HaCaT cells for 2 hours at 4°C. E) HPV16, F) HPV18, G)HPV31, and H) HPV45 in the presence or absence of heparin (0 µg/ml and 100 µg/ml). After attachment at 4°C, the cells were shifted to 37°C for an additional 2 to 4 hours. Analysis of the number of particles was done by SYBG q-PCR with attachment in the absence of heparin set to 100%.

The effect of heparin on total virus adsorption to HaCaT cells and the extracellular matrix was analyzed by measuring attachment at 4°C. No significant block was observed for attachment by HPV16 in the presence of heparin (figure 3A), suggesting HPV16 may attach to cells using a non-HS receptor. HPV18 attachment was completely blocked by heparin (figure 3B), supporting that the first step in the infectious pathway by HPV18 is to bind to HS.

Despite resistance to inhibition of HPV31 and HPV45 infection in the presence of heparin (figure 3C, D), a nearly complete block of attachment was observed for both virus types in the presence of heparin (figure 3C, D). After binding to cells, HPV virus particles have been shown to be surface bound for several hours [25], [36]–[38] and conformational changes of surface-bound papillomavirus particles are well documented [8], [25], [39]. It has been suggested that conformational changes are responsible for the slow entry kinetics of the virus particles [36]. Thus, heparin, despite not being able to block infection may still bind to the particles and prevent efficient conformational changes thus preventing initial attachment to cells. We tested this by chasing attachment for 2 hours at 4°C with increased times at 37°C. HPV16 and HPV18 attachment in the presence of heparin did not change over time (figure 3E, F). For HPV31, switching the cells to 37°C allowed for an increased number of particles to adhere to the cells in the presence of heparin over time (figure 3G), suggesting that coating of the particles by heparin at 4°C may present a block to conformational changes that need to take place. HPV45 attachment in the presence of heparin remained blocked, even up to 8 hrs at 37°C (figure 3H and data not shown). Initial HPV45 receptor-binding may be of very low affinity and/or HPV45 transfer to a higher affinity entry receptor may require more time.

### Effects of Chondroitin Sulfate

**Figure 4:**
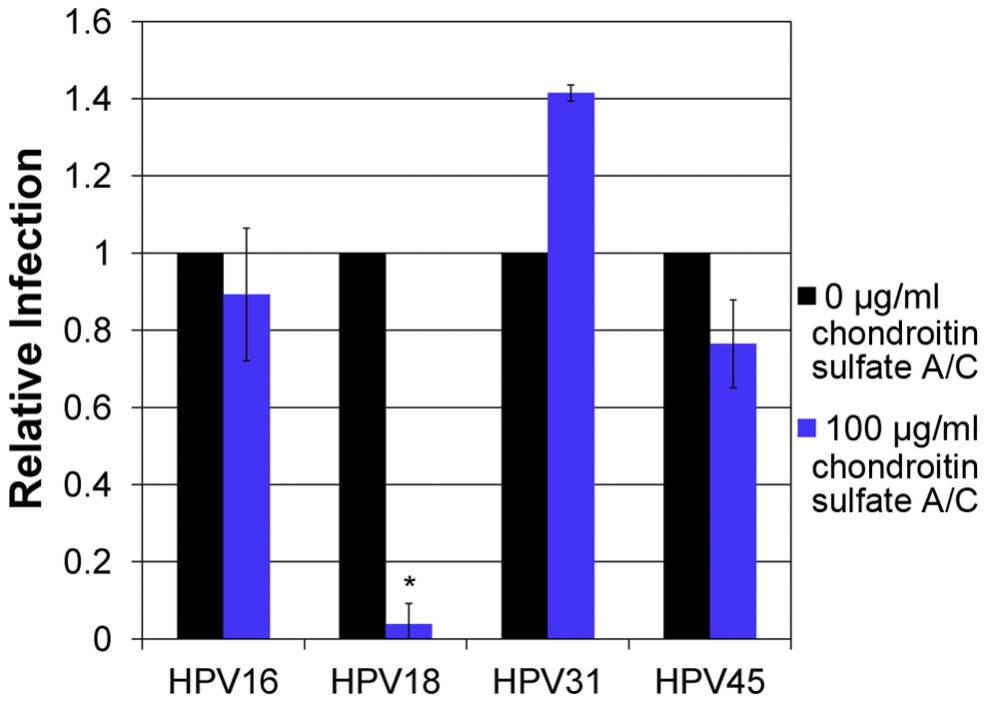
Inhibitory Effects of Chondroitin A/C against various HPV types. HPV16, HPV18, HPV31, and HPV45 were incubated with or without chondroitin A/C (0 µg/ml, and 100 µg/ml) for 30 min’s at 37°C prior to infection and during infection of HaCaT cells. All infections were done at an MOI of 10. Infections were analyzed by RT-qPCR measuring the relative amount of E1^E4 transcript two days post-infection. The data is plotted as relative infection at the different concentration with infection at 0 µg/ml of chondroitin A/C set equal to one.

Two main categories of GAGs, glucosaminoglycans, including HS, and galactosaminoglycans, including chondroitin sulfate (CS), exist. To examine the possibility of HPV interactions with different GAG types, we investigated the role of CS for infection by adding chondroitin A/C exogenously during infection (figure 4). Only HPV18 was sensitive to the addition of CS, whereas HPV16, HPV31, and HPV45 were resistant. The sensitivity of HPV18 to both heparin and CS suggests that the determinant for attachment is a combination of the type of GAG and/or GAG sulfate modifications.

### Infection in Absence of Sulfate Modifications

**Figure 5:**
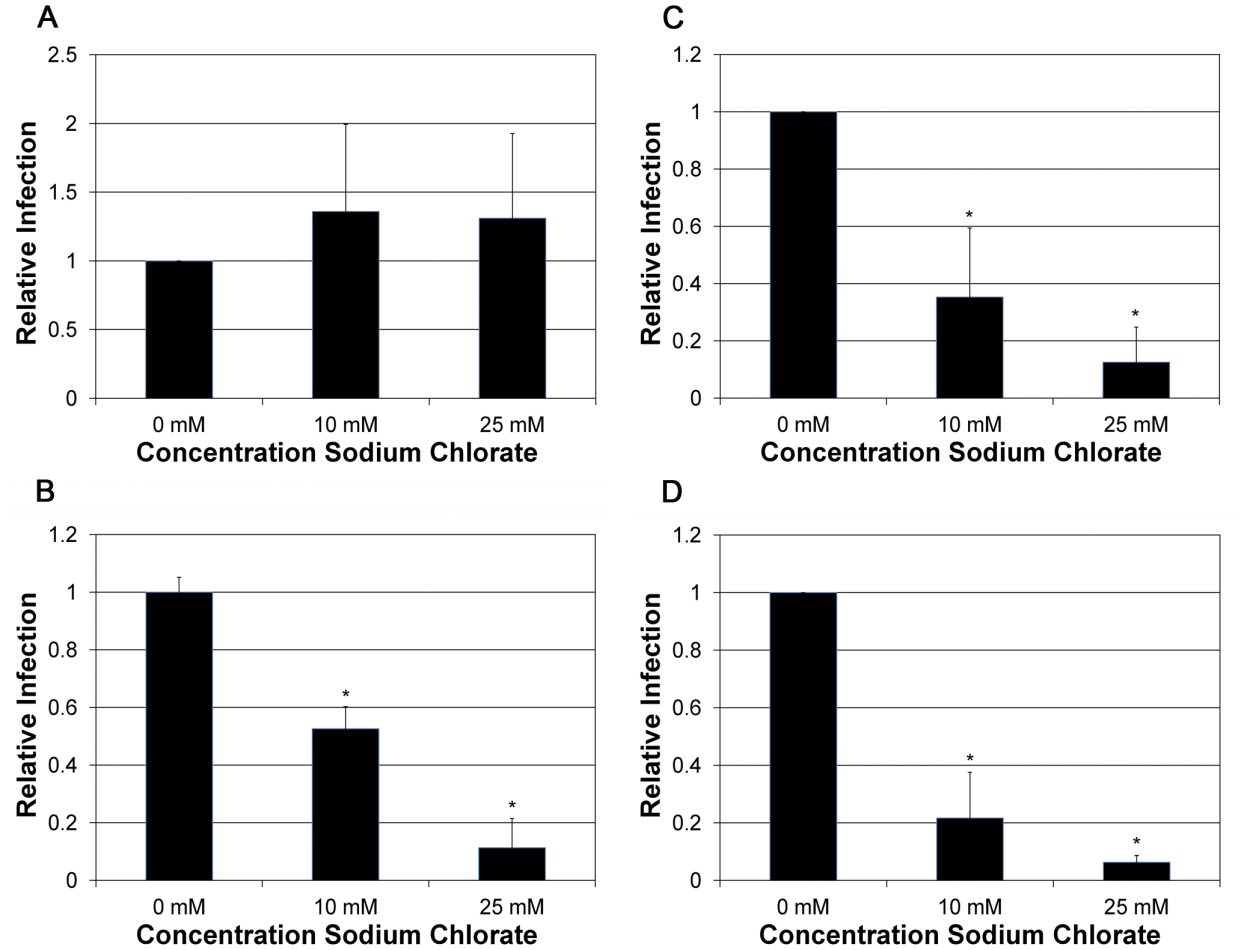
Infection of sodium chlorate-treated HaCaT cells. HaCaT cells were treated with increasing concentrations of sodium chlorate (0 mM, 10 mM and 25 mM) 2 days prior to infection and during infection by A) HPV16, B) HPV18, C)HPV31, and D) HPV45 at an MOI of 10. Infections were analyzed by RT-qPCR measuring the relative amount of E1^E4 transcript two days post-infection. The data is plotted as relative infection at the different concentration with infection at 0 mM sodium chlorate set equal to one.

The sulfation patterns of GAGs have been shown to play a role in the ability to inhibit HPV PsV infection [40]. To examine infection of NV particles in cells deficient in cell surface GAG sulfate modifications, we treated HaCaT cells with increasing concentrations of sodium chlorate. HPV16 efficiently infected sodium chlorate-treated cells (figure 5A), suggesting HPV16 does not depend on GAG sulfate modifications for infection. In contrast, HPV18 was efficiently blocked (figure 5B). HPV31 and HPV45 were also inhibited by the absence of GAG sulfation in a dose-dependent manner (figure 5C, D). Given that HPV31 and HPV45 infections are resistant to both heparin and chondroitin during infection, this supports a requirement for GAG sulfation by these virus types during infection that may display a different GAG specificity.

### Attachment and Infection in the Absence of GAGs

**Figure 6:**
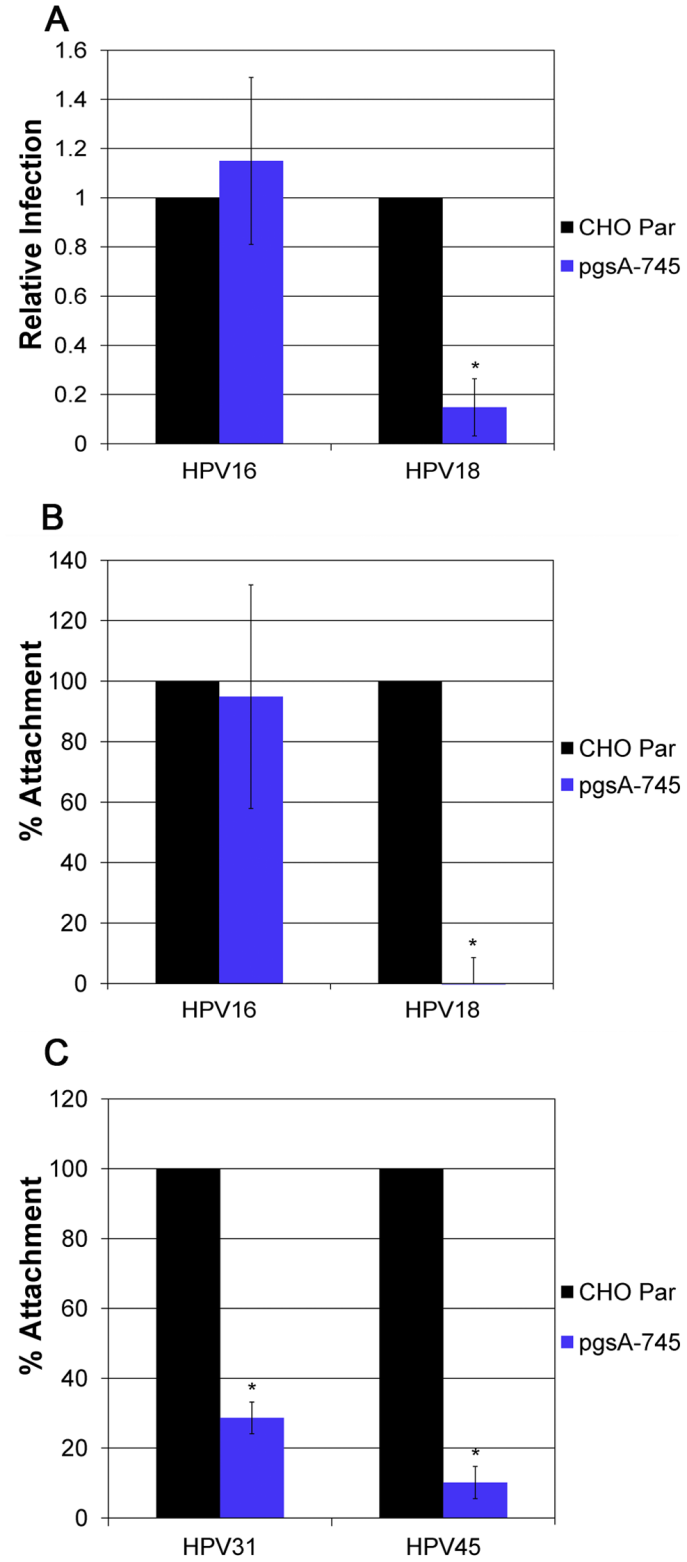
Attachment and infection in CHO parental cells and GAG-negative pgsA-745 cells. CHO Par and pgsA-745 cells were plated 2 days prior to infection by A) HPV16 and HPV18 at an MOI of 10 or attachment by B) HPV16 and HPV18, and D) HPV31 and HPV45. Infections were analyzed by RT-qPCR measuring the relative amount of E1^E4 transcript two days post-infection normalizing to infection by the CHO Par cells. Analysis of the number of particles was done by SYBG q-PCR after incubating for 2 hours at 4°C, normalizing attachment to the CHO Par cells.

To further analyze the specificity of NV HPV types for GAG binding, we took advantage of CHO cells either expressing or deficient for the expression of GAGs. We found that GAG-deficient pgsA-745 cells were infected by HPV16 at similar levels compared to CHO parental cells (figure 6A). Further, HPV16 binding to pgsA-745 cells was equivalent to that of CHO parental cells (figure 6B), supporting the ability of HPV16 to infect cells in a GAG-independent manner. In contrast, HPV18 infection was compromised in pgsA-745 cells (figure 6A). A decrease in HPV18 binding added to the evidence for a requirement of GAGs for primary attachment and infection (figure 6B).

Infection of CHO cells by HPV31 and HPV45 was too low to reproducibly detect in RT-qPCR infectivity assays. However, binding by HPV31 and HPV45, was consistently lower in the pgsA-745 cells compared to parental cells (figure 6C), suggesting that cell surface binding by HPV31 and HPV45 is dependent on cellular GAGs.

**Table 1:** GAG Dependence during Infection.

	Neutralized	Heparin	Chondroitin	Sod. Chl.	CHO +/− GAGs	Carrageenan (HaCat)	Carrageenan (HFKs)
**HPV16**	Yes	No	No	No	No	No	No
**HPV18**	Yes	Yes	Yes	Yes	Yes	Yes	Yes
**HPV31**	Yes	No	No	Yes	Nd	Yes	Yes
**HPV45**	Yes	No	No	Yes	Nd	No	No

Summary of the sensitivity of the different HPV types to neutralization by monoclonal antibodies, various polysaccharide compounds, sodium chlorate treatment, and GAG-positive or negative cells. Nd = not done.

### Differential Susceptibilities to Carrageenan in HaCaT cells

**Figure 7:**
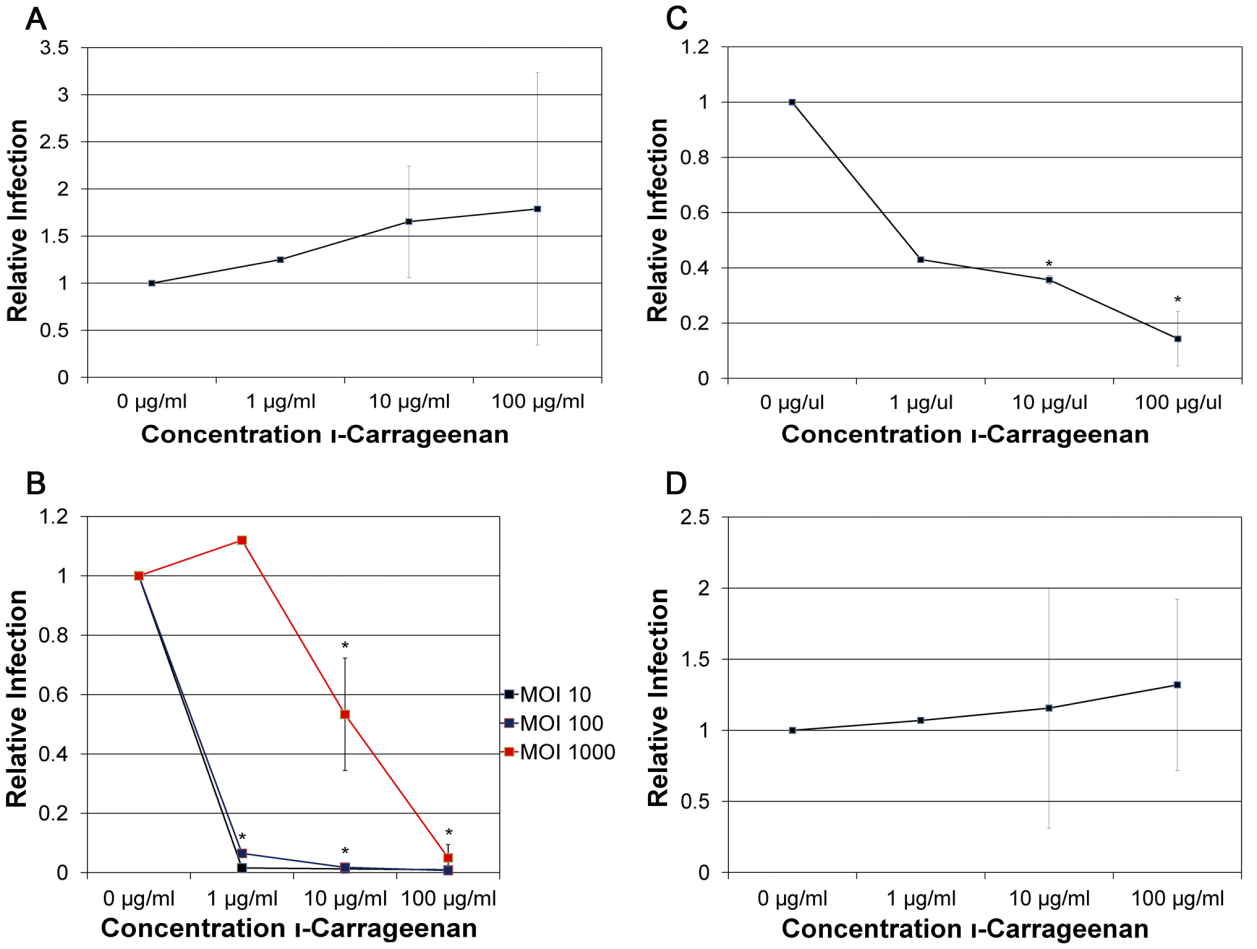
Antimicrobial effects byι-carrageenan on infection by native HPV particles. Infections of HaCaT cells were performed with A) HPV16, B) HPV18, C)HPV31, and D) HPV45 at an MOI of 10 for all virus types as well as an MOI of 100 and 1000 for HPV18. Virus was incubated with ι-carrageenan at increasing concentrations (0 µg/ml, 1 µg/ml, 10 µg/ml, and 100 µg/ml) at 37°C for 30 min prior to and during infection. Infections were analyzed by RT-qPCR measuring the relative amount of E1^E4 transcript two days post-infection. The data is plotted as relative infection at the different concentration with infection at 0 µg/ml of carrageenan set equal to one.

An *in vitro* screen of compounds that can effectively block infection by high-risk HPV PsV identified carrageenan, a highly sulfated polysaccharide derived from red algae, as a powerful inhibitor [13]. In contrast, for NV at an MOI of 10, carrageenan failed to inhibit infection by HPV16 at concentrations up to 100 µg/ml (figure 7A). For HPV18, when an MOI of 10 and 100 were used, significant levels of inhibition were observed at 1 µg/ml (figure 7B). When the titer was increased to an MOI of 1000, 50% inhibition increased to 10 µg/ml (figure 7B). In contrast, a 1000-fold lower IC_50_ in the ng/ml range was observed for various HPV PsV types [13], suggesting that the NV is more resistant to inhibition than PsV. Infection by HPV31 was also sensitive to inhibition by carrageenan (figure 7C), in contrast to the observed resistance to heparin and CS. This suggests a very selective requirement for a specific type of sulfated GAG for HPV31 infection. HPV45 did not show a dose-dependent decrease in infection in the presence of carrageenan (figure 7D), suggesting yet another preference for a different type of sulfated GAG.

**Figure 8:**
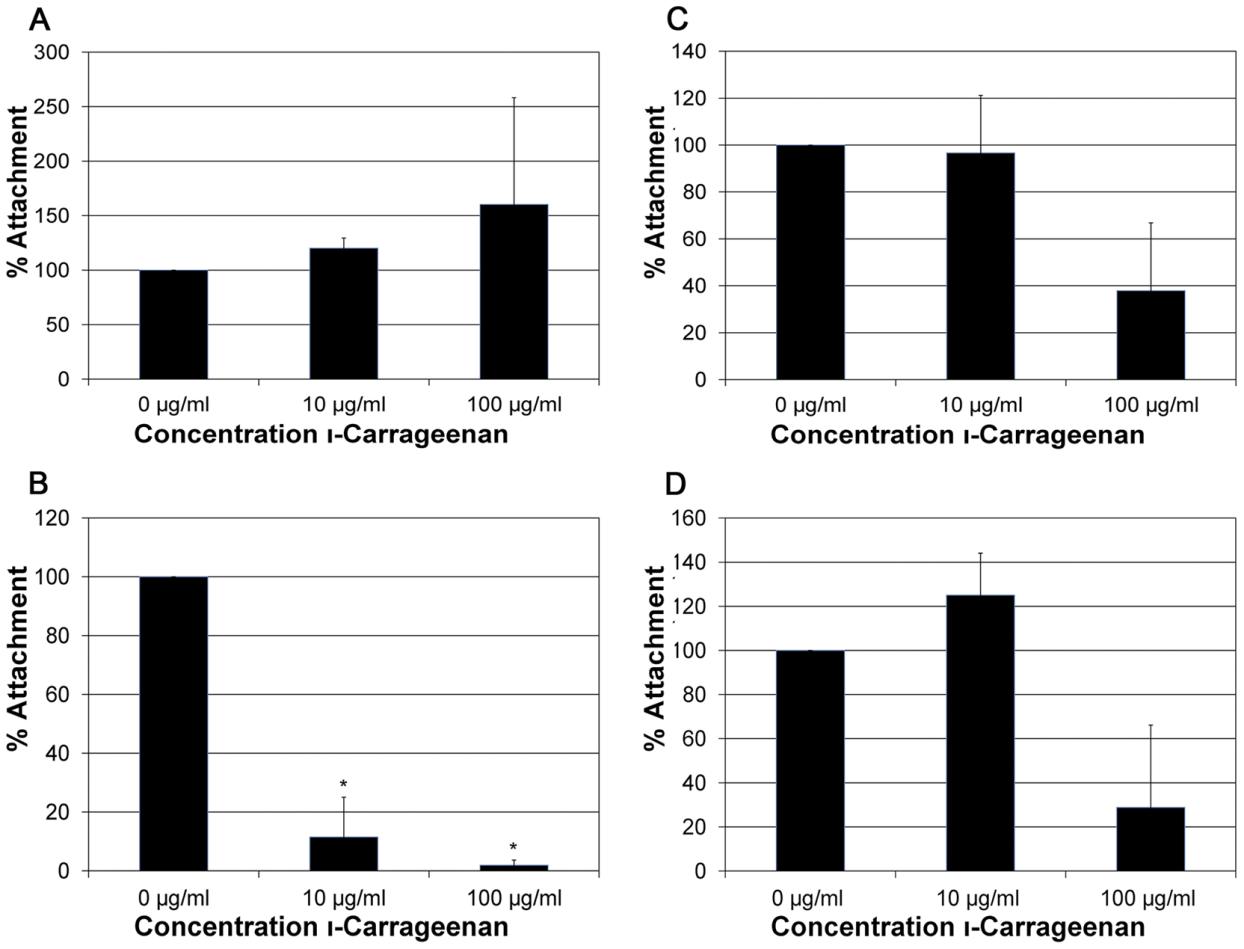
Effect ofι-carrageenan on virus adsorption. A) HPV16, B) HPV18, C)HPV31, and D) HPV45 were incubated in the presence or absence of carrageenan (0 µg/ml and 100 µg/ml) and added to HaCaT cells at an MOI of 25. The virus was allowed to attach to HaCaT cells for 2 hours at 4°C. Analysis of the number of particles was done by SYBG q-PCR with attachment in the absence of carrageenan set to 100%.

We then analyzed virion attachment in the presence of increasing concentrations of carrageenan. HPV16 was not affected at the level of attachment (figure 8A). HPV18 attachment was completely blocked by carrageenan (figure 8B). HPV31 attachment was not as sensitive (figure 8C) as to account for the level of inhibition observed during infection (figure 7C). This suggests that carrageenan might have an additional post-attachment inhibitory effect on HPV31. HPV45 attachment in the presence of carrageenan was also not significantly reduced (figure 8D).

### Antiviral Activities by Carrageenan in Primary Cells

**Figure 9:**
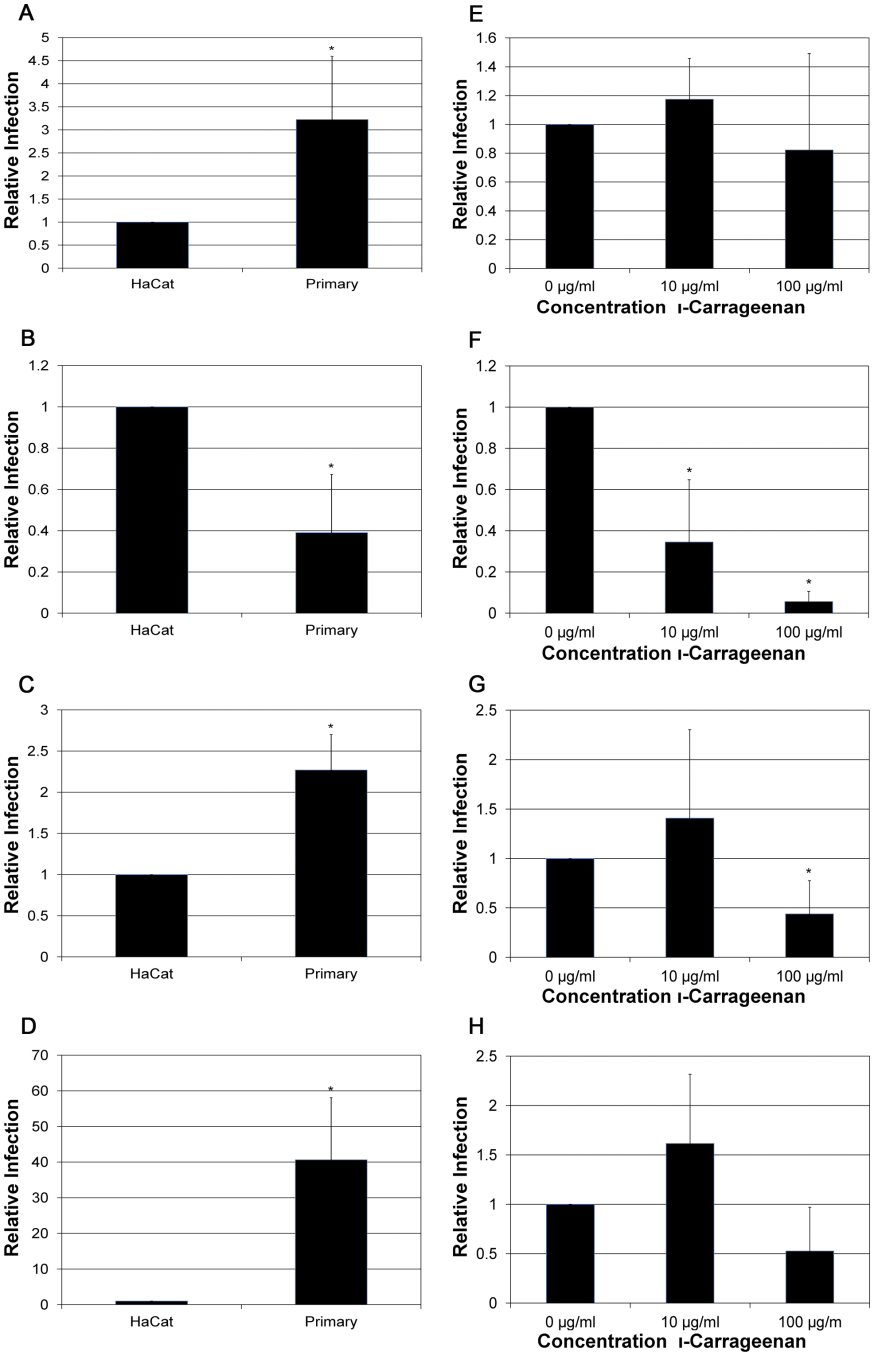
Infection of primary cells and the inhibitory effects of carrageenan on the infection of primary cells. HaCaT and primary cells were infected at an MOI of 10 by A) HPV16, B) HPV18, C) HPV31, and D) HPV45. Infections of primary cells were performed in the presence ι-carrageenan at increasing concentrations (0 µg/ml, 10 µg/ml, and 100 µg/ml) for; E) HPV16, F) HPV18, G)HPV31, and H) HPV45. Infections were analyzed by RT-qPCR measuring the relative amount of E1^E4 transcript two days post-infection. The data is plotted as relative infection at the different concentration with infection at 0 µg/ml of carrageenan set equal to one.

We used primary cells in culture to verify key data generated using HaCaT keratinocytes. As PsV infection of primary cells in culture has been shown to be very inefficient [27], we first wanted to establish the ability to infect primary keratinocytes with NV. Infections by HPV16, HPV31, and HPV45 were comparable to, or better than, infection of HaCaT keratinocytes (figure 9A, C, D). In contrast, HPV18 infection is reproducibly weaker in primary keratinocytes (figure 9B).

We next investigated the efficiency of inhibition by carrageenan against infection by the different high-risk HPV types in primary keratinocytes. The lack of a dose-dependent inhibition of HPV16 infection of primary cells supports that HPV16 NV is insensitive to inhibition by carrageenan (figure 9E). An MOI of 100, instead of 10, was used for HPV18 infections, as this resulted in infection levels similar to that observed in HaCaT cells (data not shown). Infection by HPV18 was blocked by carrageenan, (figure 9F), supporting the need for GAGs as primary attachment receptors for HPV18. HPV31 was not significantly blocked in the presence of carrageenan at 10 µg/ml, but was blocked only at the higher concentration of 100 µg/ml (figure 9G). Similarly to that seen in HaCaT cells, HPV45 infection of primary keratinocytes was not significantly blocked by carrageenan (figure 9H).

## Discussion

As summarized in Table I, a complicated pattern of dependency on cell surface GAGs and/or GAG modifications, emerges from the comparison of infection by the four main cancer-causing HPV types, HPV16, HPV18, HPV31, and HPV45. We show here that raft-derived HPV18 requires HS binding for infection, similar to previously reported data using recombinant HPV particles. Noticeably, HPV18 infection is also sensitive to CS, whereas infections by HPV16 and HPV18 PsVs, are not substantially inhibited by the presence of CS [23], [41].

HPV31 and HPV45 display a different pattern of inhibition under our experimental conditions. The reduction in infectivity after sodium chlorate treatment of the cells, and the inability to attach to GAG-deficient cells, suggests a preference for a different type of sulfated GAG. Previous research has shown that the type and level of sulfation may play an important role for the interaction of PsV with polysaccharides [23], [40]. Interestingly, despite not blocking infection, heparin effectively blocked attachment by HPV31 and HPV45. It is possible that despite not being able to block infection, heparin still attaches to the particles. HPV5 PsV has been shown to bind carrageenan beads even though no block was observed at the level of infection [13]. In a recent study attachment to the cell surface but not the ECM was blocked by heparin pre-incubated HPV16 PsV [23]. Further, it was demonstrated that bound heparin conferred a conformational change to the particles [23]. Thus, it is possible that residual levels of heparin-coated virus binding to the ECM are able to transfer directly to a secondary entry receptor on the cell surface.

Unexpectedly, native HPV16 does not follow the same mode of dependence on GAGs for attachment. Binding to GAGs is not required for attachment or infection by HPV16, as shown by incubation with soluble heparin, the ability of HPV16 to infect GAG-deficient cells, and by preventing sulfate modifications of GAGs by sodium chlorate. In addition, PsV do not efficiently infect primary keratinocytes in culture [27], which was hypothesized to be due to altered structure of HS modifications during *in vitro* culturing of keratinocytes [42]. Interestingly, infection of primary keratinocytes by HPV18 NV is also inefficient, whereas primary keratinocytes are readily infected by HPV16, HPV31, and HPV45 NV. Together, these results suggest that different HPV types have distinct requirements for entry into their natural host cells. Further research is required to determine whether the GAG-dependent and independent HPV types post-attachment share the same receptor for internalization into their natural host cells. Interestingly, a recent publication demonstrated that HPV particles may be released from the cell surface in complex with HS, followed by re-attachment to the cell surface independent of cell surface GAGs, to initiate infection [29]. Additional studies are needed to determine the composition of native particles as they are released from the cells and whether that plays an important role in HPV NV infection.

The observed differences in GAG-dependency between different HPV types, as well as between PsV and NV particles, are unlikely to be explained simply due to the use of different cell lines and different requirements for *in vivo* and *in vitro* infections. Using the same HaCaT keratinocyte cell line, as used in previous PsV infection studies [13], [41], we found that the sensitivity to polysaccharide compounds is greatly reduced or completely lacking for native HPV particles. It has been shown that the need for initial attachment to HS is tightly correlated with the need for cleavage by cellular furin during infection, where furin pre-cleaved PsV particles can bypass binding to HS for infection [27]. Bypassing HS-binding could allow for direct binding to a functional entry receptor. It is possible that HPV16, which does not need HS binding during infection, is pre-cleaved during virion morphogenesis. It is also conceivable that different HPV species utilize different molecules for initial attachment to the basement membrane and the host cell. Laminin-332 has been shown to be the preferential receptor for HPV11 and HPV16 PsV for initial attachment [22], [23]. Studies using PsV show that different types of HPV may utilize different entry pathways [20], suggesting that binding to different receptor molecules on the cell surface may direct these viruses into different endocytic pathways. Moreover, although not required, it is still possible that HPV16 interacts with GAGs through conserved motifs on the virus surface [43]. *In vivo*, this may serve to concentrate the virus to the site of infection.

Importantly, neutralization by carrageenan was shown to be effective against HPV18 and HPV31 infections. However, HPV16 and HPV45 could not be blocked by carrageenan, suggesting carrageenan may not protect against all HPV types. Inhibition of dengue virus infection by carrageenan also showed differential efficacy depending on the serotype tested [44]. Different carrageenan compounds need to be considered, as a preparation of more than one type of carrageenan might be able to protect against more HPV types [13], [44].

The efficient production of synthetic particles is invaluable for rapid analysis of many aspects of the papillomavirus life cycle. However, a complete comparison of the structure of PsV and NV has not been published and experimental results using PsV particles as a surrogate need to be verified with NV [45]–[47]. As described here, genital HPVs may use different strategies to attach to and infect their host. As a result, they may naturally exhibit differential susceptibility to various agents, an important factor to consider when developing new agents to block HPV transmission.

## Methods

### Ethics Statement

The use of human cervical and foreskin keratinocyte tissues to develop cell lines as well as for infectivity assays for these studies was approved by the Institutional review Board at the Pennsylvania State University College of Medicine and by the Institutional review Board at Pinnacle Health Hospitals. Discarded, de-identified tissues were exempt from needing informed patient consent. Informed consent was waived by both Institutional Review Boards.

### Cell Culture and Virus Production

HaCaT cells were maintained in DMEM supplemented with 10% FBS, 0.025 mg/ml Gentamicin, and 0.11 mg/ml sodium pyruvate. CHO cells were maintained in minimal essential α-medium supplemented with 10% FBS and antibiotics. Primary human keratinocytes from newborn foreskin circumcision and cervical biopsies were isolated as previously described [16]. Keratinocytes were maintained in 154 medium supplemented with Human Keratinocyte Growth Supplement Kit (Cascade Biologics, Inc., Portland, OR). Immortalized keratinocytes stably maintaining HPV episomes were cultured in E-medium with J2 3T3 feeder cells [18] and grown in raft culture to produce virus as previously described [18]. Mature virus particles were harvested from tissues after 20 days [17].

Rafts were harvested and virus was isolated by homogenization in phosphate buffer (.005 M Na-phosphate, pH 8, 2 mM MgCl_2_) as previously described [17]. All virus preps for titering and infectivity assays were treated with benzonase (375 U) at 37°C for one hour to remove any un-encapsidated viral genomes. Samples were adjusted to 1 M NaCl and centrifuged at 4°C for 10 minutes at 10,500 rpm to remove cellular debris.

### Virus Titers

To release the viral genomes, 10 µl of a virus prep was re-suspended in a 200 µl HIRT DNA extraction buffer (400 mM NaCl/10 mM Tris-HCl, pH 7.4/10 mM EDTA, pH 8.0), with 2 µl 20 mg/ml Proteinase K, and 10 µl 10% SDS for 2 hours at 37°C. The DNA was purified by phenol-chloroform extraction followed by ethanol precipitation and re-suspended in 20 µl TE [17]. Titers were determined using a qPCR-based DNA encapsidation assay utilizing a Qiagen Quantitect SYBR Green PCR Kit. Amplification of the viral genome target was performed using previously described E2 primers against a standard curve of 10-fold serial dilutions from 10^8^ to 10^4^ copies per µl [17], [35].

### Infections, Neutralizations, and Inhibition Assays

The polysaccharides heparin (H4784, high molecular weight) and chondroitin sulfate A/C (C4384), sodium chlorate (403016) and ι-carrageenan (C4014) were purchased from Sigma-Aldrich. The anti-L1 antibodies were a gift from Neil D Christensen. H16.V5, H18.J4, H31.A6, and H45.N5 were used to neutralize HPV16, HPV18, HPV31, and HPV45, respectively. HaCaT cells were seeded in 24-well plates, 50,000 cells per well 2 days prior to infection. CHO cells were seeded 30,000 per well and healthy low-passage primary cells were seeded 70,000 per well. Compounds were mixed with virus and media in a total volume of 500 µl prior to addition to cells. An MOI of 10 particles per cell was used unless otherwise noted. For sodium chlorate, cells were grown in the presence of the compound 2 days prior to and during the infection. Virus was incubated with the cells for 48 hrs at 37°C and mRNA was harvested using a Qiagen RNAeasy Kit. Infection was analyzed using a previously described RT-qPCR-based infectivity assaying for E1^E4 transcript levels [17], [35]. Results are representative of means and standard deviations for at least three independent infections from at least 2 different batches of virus preps for each virus type. Students t-test was performed with statistical significance calculated (p<0.05).

### Attachment Assays

Cells were seeded the same way as in the infectivity assays. The virus was incubated with the cells at an MOI of 25 for 2 hours at 4°C with or without compound, and then shifted after the indicated number of hours to 37°C. Cells were washed 3x with PBS before lysing on the plate for 2 hours at 37°C using a HIRT lysis buffer, followed by phenol-chloroform extraction and ethanol precipitation. The number of particles attached to the cells was determined as the number of viral genomes by amplifying a target sequence in the E2 gene, as in the DNA encapsidation assay. Results are representative of means and standard deviations for at least three independent attachments assays from at least 2 different virus batches. Students t-test was performed with statistical significance calculated (p<0.05).

## References

[1] Schiffman M, Kjaer SK (2003) Chapter 2: Natural history of anogenital human papillomavirus infection and neoplasia. J Natl Cancer Inst Monogr: 14–19. 10.1093/oxfordjournals.jncimonographs.a003476 12807940

[2] zur Hausen H (2009) Papillomaviruses in the causation of human cancers - a brief historical account. Virology 384: 260–265. 19135222 10.1016/j.virol.2008.11.046

[3] Giroglou T, Florin L, Schafer F, Streeck RE, Sapp M (2001) Human papillomavirus infection requires cell surface heparan sulfate. J Virol 75: 1565–1570. 11152531 10.1128/JVI.75.3.1565-1570.2001 PMC114064

[4] Culp TD, Budgeon LR, Christensen ND (2006) Human papillomaviruses bind a basal extracellular matrix component secreted by keratinocytes which is distinct from a membrane-associated receptor. Virology 347: 147–159. 16376962 10.1016/j.virol.2005.11.025

[5] Pereira R, Hitzeroth, II, Rybicki EP (2009) Insights into the role and function of L2, the minor capsid protein of papillomaviruses. Arch Virol 154: 187–197. 19169853 10.1007/s00705-009-0310-3

[6] Finnen RL, Erickson KD, Chen XS, Garcea RL (2003) Interactions between papillomavirus L1 and L2 capsid proteins. J Virol 77: 4818–4826. 12663788 10.1128/JVI.77.8.4818-4826.2003 PMC152166

[7] Zhao KN, Sun XY, Frazer IH, Zhou J (1998) DNA packaging by L1 and L2 capsid proteins of bovine papillomavirus type 1. Virology 243: 482–491. 9568045 10.1006/viro.1998.9091

[8] Yang R, Day PM, Yutzy WHt, Lin KY, Hung CF, et al (2003) Cell surface-binding motifs of L2 that facilitate papillomavirus infection. J Virol 77: 3531–3541. 12610128 10.1128/JVI.77.6.3531-3541.2003 PMC149523

[9] Yang R, Yutzy WHt, Viscidi RP, Roden RB (2003) Interaction of L2 with beta-actin directs intracellular transport of papillomavirus and infection. J Biol Chem 278: 12546–12553. 12560332 10.1074/jbc.M208691200

[10] Kamper N, Day PM, Nowak T, Selinka HC, Florin L, et al (2006) A membrane-destabilizing peptide in capsid protein L2 is required for egress of papillomavirus genomes from endosomes. J Virol 80: 759–768. 16378978 10.1128/JVI.80.2.759-768.2006 PMC1346844

[11] Woodham AW, Da Silva DM, Skeate JG, Raff AB, Ambroso MR, et al (2012) The S100A10 subunit of the annexin A2 heterotetramer facilitates L2-mediated human papillomavirus infection. PLoS One 7: e43519. 22927980 10.1371/journal.pone.0043519 PMC3425544

[12] Doorbar J (2006) Molecular biology of human papillomavirus infection and cervical cancer. Clin Sci (Lond) 110: 525–541. 16597322 10.1042/CS20050369

[13] Buck CB, Thompson CD, Roberts JN, Muller M, Lowy DR, et al (2006) Carrageenan is a potent inhibitor of papillomavirus infection. PLoS Pathog 2: e69. 16839203 10.1371/journal.ppat.0020069 PMC1500806

[14] Luciani S, Jauregui B, Kieny C, Andrus JK (2009) Human papillomavirus vaccines: new tools for accelerating cervical cancer prevention in developing countries. Immunotherapy 1: 795–807. 20636024 10.2217/imt.09.48

[15] Meyers C, Mayer TJ, Ozbun MA (1997) Synthesis of infectious human papillomavirus type 18 in differentiating epithelium transfected with viral DNA. J Virol 71: 7381–7386. 9311816 10.1128/jvi.71.10.7381-7386.1997 PMC192083

[16] McLaughlin-Drubin ME, Wilson S, Mullikin B, Suzich J, Meyers C (2003) Human papillomavirus type 45 propagation, infection, and neutralization. Virology 312: 1–7. 12890615 10.1016/s0042-6822(03)00312-x

[17] Conway MJ, Alam S, Ryndock EJ, Cruz L, Christensen ND, et al (2009) Tissue-spanning redox gradient-dependent assembly of native human papillomavirus type 16 virions. J Virol 83: 10515–10526. 19656879 10.1128/JVI.00731-09 PMC2753102

[18] Meyers C, Frattini MG, Hudson JB, Laimins LA (1992) Biosynthesis of human papillomavirus from a continuous cell line upon epithelial differentiation. Science 257: 971–973. 1323879 10.1126/science.1323879

[19] Buck CB, Pastrana DV, Lowy DR, Schiller JT (2004) Efficient intracellular assembly of papillomaviral vectors. J Virol 78: 751–757. 14694107 10.1128/JVI.78.2.751-757.2004 PMC368835

[20] Sapp M, Bienkowska-Haba M (2009) Viral entry mechanisms: human papillomavirus and a long journey from extracellular matrix to the nucleus. Febs J 276: 7206–7216. 19878308 10.1111/j.1742-4658.2009.07400.x PMC2795018

[21] Kines RC, Thompson CD, Lowy DR, Schiller JT, Day PM (2009) The initial steps leading to papillomavirus infection occur on the basement membrane prior to cell surface binding. Proc Natl Acad Sci U S A 106: 20458–20463. 19920181 10.1073/pnas.0908502106 PMC2787115

[22] Culp TD, Budgeon LR, Marinkovich MP, Meneguzzi G, Christensen ND (2006) Keratinocyte-secreted laminin 5 can function as a transient receptor for human papillomaviruses by binding virions and transferring them to adjacent cells. J Virol 80: 8940–8950. 16940506 10.1128/JVI.00724-06 PMC1563898

[23] Cerqueira C, Liu Y, Kuhling L, Chai W, Hafezi W, et al.. (2013) Heparin increases the infectivity of Human Papillomavirus Type 16 independent of cell surface proteoglycans and induces L1 epitope exposure. Cell Microbiol. 10.1111/cmi.12150 PMC4731924 23601855

[24] Richards RM, Lowy DR, Schiller JT, Day PM (2006) Cleavage of the papillomavirus minor capsid protein, L2, at a furin consensus site is necessary for infection. Proc Natl Acad Sci U S A 103: 1522–1527. 16432208 10.1073/pnas.0508815103 PMC1360554

[25] Day PM, Gambhira R, Roden RB, Lowy DR, Schiller JT (2008) Mechanisms of human papillomavirus type 16 neutralization by l2 cross-neutralizing and l1 type-specific antibodies. J Virol 82: 4638–4646. 18305047 10.1128/JVI.00143-08 PMC2293042

[26] Selinka HC, Florin L, Patel HD, Freitag K, Schmidtke M, et al (2007) Inhibition of transfer to secondary receptors by heparan sulfate-binding drug or antibody induces noninfectious uptake of human papillomavirus. J Virol 81: 10970–10980. 17686860 10.1128/JVI.00998-07 PMC2045555

[27] Day PM, Lowy DR, Schiller JT (2008) Heparan sulfate-independent cell binding and infection with furin-precleaved papillomavirus capsids. J Virol 82: 12565–12568. 18829767 10.1128/JVI.01631-08 PMC2593329

[28] Evander M, Frazer IH, Payne E, Qi YM, Hengst K, et al (1997) Identification of the alpha6 integrin as a candidate receptor for papillomaviruses. J Virol 71: 2449–2456. 9032382 10.1128/jvi.71.3.2449-2456.1997 PMC191355

[29] Surviladze Z, Dziduszko A, Ozbun MA (2012) Essential roles for soluble virion-associated heparan sulfonated proteoglycans and growth factors in human papillomavirus infections. PLoS Pathog 8: e1002519. 22346752 10.1371/journal.ppat.1002519 PMC3276557

[30] Bienkowska-Haba M, Patel HD, Sapp M (2009) Target cell cyclophilins facilitate human papillomavirus type 16 infection. PLoS Pathog 5: e1000524. 19629175 10.1371/journal.ppat.1000524 PMC2709439

[31] Patterson NA, Smith JL, Ozbun MA (2005) Human papillomavirus type 31b infection of human keratinocytes does not require heparan sulfate. J Virol 79: 6838–6847. 15890923 10.1128/JVI.79.11.6838-6847.2005 PMC1112118

[32] Bauer PH, Cui C, Liu WR, Stehle T, Harrison SC, et al (1999) Discrimination between sialic acid-containing receptors and pseudoreceptors regulates polyomavirus spread in the mouse. J Virol 73: 5826–5832. 10364334 10.1128/jvi.73.7.5826-5832.1999 PMC112643

[33] Sapp M, Day PM (2009) Structure, attachment and entry of polyoma- and papillomaviruses. Virology 384: 400–409. 19157477 10.1016/j.virol.2008.12.022

[34] Neu U, Maginnis MS, Palma AS, Stroh LJ, Nelson CD, et al (2010) Structure-function analysis of the human JC polyomavirus establishes the LSTc pentasaccharide as a functional receptor motif. Cell Host Microbe 8: 309–319. 20951965 10.1016/j.chom.2010.09.004 PMC2957469

[35] Conway MJ, Cruz L, Alam S, Christensen ND, Meyers C (2011) Cross-neutralization potential of native human papillomavirus N-terminal L2 epitopes. PLoS One 6: e16405. 21346798 10.1371/journal.pone.0016405 PMC3035607

[36] Schelhaas M, Shah B, Holzer M, Blattmann P, Kuhling L, et al (2012) Entry of human papillomavirus type 16 by actin-dependent, clathrin- and lipid raft-independent endocytosis. PLoS Pathog 8: e1002657. 22536154 10.1371/journal.ppat.1002657 PMC3334892

[37] HIV/AIDS and STD Updates. AIDS Patient Care STDS 25: 265–267.

[38] Smith JL, Campos SK, Ozbun MA (2007) Human papillomavirus type 31 uses a caveolin 1- and dynamin 2-mediated entry pathway for infection of human keratinocytes. J Virol 81: 9922–9931. 17626097 10.1128/JVI.00988-07 PMC2045393

[39] Selinka HC, Giroglou T, Nowak T, Christensen ND, Sapp M (2003) Further evidence that papillomavirus capsids exist in two distinct conformations. J Virol 77: 12961–12967. 14645552 10.1128/JVI.77.24.12961-12967.2003 PMC296061

[40] Lembo D, Donalisio M, Rusnati M, Bugatti A, Cornaglia M, et al (2008) Sulfated K5 Escherichia coli polysaccharide derivatives as wide-range inhibitors of genital types of human papillomavirus. Antimicrob Agents Chemother 52: 1374–1381. 18250186 10.1128/AAC.01467-07 PMC2292566

[41] Johnson KM, Kines RC, Roberts JN, Lowy DR, Schiller JT, et al (2009) Role of heparan sulfate in attachment to and infection of the murine female genital tract by human papillomavirus. J Virol 83: 2067–2074. 19073722 10.1128/JVI.02190-08 PMC2643729

[42] Turnbull J, Powell A, Guimond S (2001) Heparan sulfate: decoding a dynamic multifunctional cell regulator. Trends Cell Biol 11: 75–82. 11166215 10.1016/s0962-8924(00)01897-3

[43] Dasgupta J, Bienkowska-Haba M, Ortega ME, Patel HD, Bodevin S, et al (2010) Structural basis of oligosaccharide receptor recognition by human papillomavirus. J Biol Chem 286: 2617–2624. 21115492 10.1074/jbc.M110.160184 PMC3024757

[44] Talarico LB, Damonte EB (2007) Interference in dengue virus adsorption and uncoating by carrageenans. Virology 363: 473–485. 17337028 10.1016/j.virol.2007.01.043

[45] Sapp M, Fligge C, Petzak I, Harris JR, Streeck RE (1998) Papillomavirus assembly requires trimerization of the major capsid protein by disulfides between two highly conserved cysteines. J Virol 72: 6186–6189. 9621087 10.1128/jvi.72.7.6186-6189.1998 PMC110432

[46] Fligge C, Schafer F, Selinka HC, Sapp C, Sapp M (2001) DNA-induced structural changes in the papillomavirus capsid. J Virol 75: 7727–7731. 11462046 10.1128/JVI.75.16.7727-7731.2001 PMC115009

[47] Buck CB, Thompson CD, Pang YY, Lowy DR, Schiller JT (2005) Maturation of papillomavirus capsids. J Virol 79: 2839–2846. 15709003 10.1128/JVI.79.5.2839-2846.2005 PMC548454

